# OMV Vaccines and the Role of TLR Agonists in Immune Response

**DOI:** 10.3390/ijms21124416

**Published:** 2020-06-21

**Authors:** Francesca Mancini, Omar Rossi, Francesca Necchi, Francesca Micoli

**Affiliations:** GSK Vaccines Institute for Global Health s.r.l (GVGH), 53100 Siena, Italy; francesca.x.mancini@gsk.com (F.M.); omar.x.rossi@gsk.com (O.R.); francesca.x.necchi@gsk.com (F.N.)

**Keywords:** outer membrane vesicle (OMV), GMMA, PAMP, TLR, vaccine

## Abstract

Outer Membrane Vesicles (OMVs) are bacterial nanoparticles that are spontaneously released during growth both in vitro and in vivo by Gram-negative bacteria. They are spherical, bilayered membrane nanostructures that contain many components found within the external surface of the parent bacterium. Naturally, OMVs serve the bacteria as a mechanism to deliver DNA, RNA, proteins, and toxins, as well as to promote biofilm formation and remodel the outer membrane during growth. On the other hand, as OMVs possess the optimal size to be uptaken by immune cells, and present a range of surface-exposed antigens in native conformation and Toll-like receptor (TLR) activating components, they represent an attractive and powerful vaccine platform able to induce both humoral and cell-mediated immune responses. This work reviews the TLR-agonists expressed on OMVs and their capability to trigger individual TLRs expressed on different cell types of the immune system, and then focuses on their impact on the immune responses elicited by OMVs compared to traditional vaccines.

## 1. Introduction

Outer Membrane Vesicles (OMVs) are 25–250 nm round shaped exosomes that are spontaneously released during growth by both pathogenic and nonpathogenic Gram-negative bacteria, especially during the end of log phase growth and at then sites of cell division [1].

One of the first reports on OMVs produced during the growth of *Escherichia coli* cells was published nearly 40 years ago by Wensink and Witholt [2] and indicated an imbalance of lipoproteins in OMVs versus outer membranes. This finding led the authors to propose a bulging model for OMV biogenesis which builds upon the presence of distinct membrane areas with limited peptidoglycan synthesis, and, therefore, an absent lipoprotein-mediated outer membrane connectivity from the underlying peptidoglycan. Since then, many scientists have worked to define an OMV biogenesis pathway, and several models have been suggested, such as the initiation of OMV production by the enrichment of membrane curvature-inducing molecules, or the accumulation of peptidoglycan fragments or misfolded proteins in the periplasmic space [3]. Recent research has concentrated on the molecular basis of OMV biosynthesis, including, for example, secretion and sorting of lipids into the OMVs or outer membranes [4,5].

The production of OMVs has been described for a great number of Gram-negative bacteria in all growth phases and in a variety of growth conditions. The production of vesicles has been further demonstrated to be linked to the bacterial stress response [6,7], providing it a physiological relevance as a protective mechanism for the removal of undesirable envelope components, or as a component of wellbeing increase during the colonization of host tissues [8]. As shown by Elhenawy et al. for *Salmonella enterica* serovar Typhimurium [9] and also recently for *Mycobacterium tuberculosis* [10,11], OMVs are released even during intracellular growth within the host cells.

OMVs have been proposed to have several biological functions such as long-distance delivery systems of specific components [12,13], and protection of the molecules embedded in their structure from dilution and degradation. OMVs can serve to collect nutrients or transfer virulence factors and toxins, thus contributing to building a suitable micro-environment for the growth or survival of the pathogen in a host [14,15,16,17,18]. It has been also demonstrated that OMVs can deplete the serum of antibodies and other bactericidal molecules by binding to their surface, as suggested for *Neisseria gonorrhoeae* OMV, thereby contributing to the serum resistance killing of bacteria [19]. For several bacteria, it has been shown that OMVs engage in the formation of biofilms, increasing survival in hosts [20], or in soil [21]. Moreover, OMVs production has been described as a bacterial defense mechanism against antibiotics, since it appears to dilute the gentamycin detrimental effects on the membranes [22]. Other studies have demonstrated the involvement of OMVs in the gene transfer between bacteria, e.g., for the transfer of antibiotic resistance by DNA [23]. Indeed, OMVs can enclose plasmids, chromosomal DNA fragments, or bacteriophage DNA [24].

Due to their biogenesis, OMVs largely reflect the structure of the outer membrane. Phospholipids are present on the membrane inner side and lipopolysaccharide (LPS) is present on the membrane outer side, mixed with outer membrane proteins and lipoproteins [25,26]. Various compounds from the periplasm, and to a lesser extent, from cytoplasm, such as proteins, RNA/DNA, and peptidoglycan, may be embedded in the OMV lumen [12,14].

OMVs represent the envelope of bacteria, with a wide range of surface bacterial antigens in their native conformation and orientation and an optimal size for being uptaken by immune cells. The simultaneous presence of several bacterial antigens, combined with the immunopotentiator effect of the Toll-like receptor (TLR) agonists naturally present on these systems, confer self-adjuvanticity properties to OMVs. These agonists or PAMPs (Pathogen-Associated Molecular Patterns), activators of Pattern Recognition Receptors (PRRs), make OMVs strong drivers of the innate immune response, which functions as the host’s first line of defense [27]. Moreover, several studies have highlighted OMVs’ ability to induce long-lasting humoral and cellular immune responses when used as vaccines [28,29,30]. Recently, the potential for inducing a strong immune response together with the simplicity of manufacture, have made OMVs an attractive platform for vaccine development. To this end, genetic manipulations have been introduced in bacteria to further enhance the in vitro blebbing, by the disruption of genes involved in processes including the synthesis of proteins linking the outer membrane and the underlying peptidoglycan layer, envelope structure, and cellular stress [31,32,33]. These blebs resulting from the hyperblebbing of genetically modified microorganisms have also been called GMMA (Generalized Modules for Membrane Antigens). The technology has been successfully applied to many pathogens including non-invasive *Salmonella* [34,35,36,37], *Neisseria meningitidis* [38], and *Shigella* serotypes, with the most advanced prototype tested up to phase 1 and 2 clinical studies [39,40,41]. Several industrial approaches using this and other [42] OMV-based technologies have been used and applied to produce OMVs from several Gram-negative bacteria.

Research over the last decade has demonstrated the application of OMVs as powerful, economical, and flexible tools for the delivery of various heterologous vaccine antigens, ranging from bacterial, to viral, parasitic, and even cancer antigens [43,44,45,46]. The antigen per se, or many of the molecules composing the OMV used as delivery system for the heterologous antigen, could act as an effective TLR-activating component, property considered as one of the key drivers for the powerful immune response elicited by OMV. In this review, we focus our attention on dissecting the roles of PAMPs and TLR agonists associated with OMVs and their impact on immune responses elicited by OMV-based vaccines.

## 2. Toll-like Receptors (TLR)

PRRs represent a wide class of receptors predominantly expressed on innate immune cells such as dendritic cells (DCs), macrophages, and neutrophils, as well as on cells not belonging to the immune system, such as fibroblast and epithelial cells [47]. They identify two classes of molecules: PAMPs, which belong to microbial pathogens, and Damage-Associated Molecular Patterns (DAMPs), which are associated with host cell damage or death. There are several classes of PRRs, such as TLRs, RIG-I-Like Receptors (RLRs), NOD-Like Receptors (NLRs), and DNA receptors (cytosolic sensors for DNA), each sensing different classes of PAMPs [48].

The class of PRRs most widely studied is represented by the TLRs, first discovered in *Drosophila* as proteins involved in embryonic development [49].

TLRs are type I membrane glycoproteins composed by an extracellular domain with Leucine Rich Repeats required for PAMP recognition [50], and a cytoplasmic Toll/interleukin-1 receptor (TIR) homology domain required for downstream signaling. TIR domain containing adaptor molecules such as MyD88, TRIF, TIRAP, and TRAM are recruited to the TIR domains of the different TLRs, thus activating several transcription factors such as NF-κB, IRF3/7, and MAP kinases, and leading to the production of pro-inflammatory cytokines (i.e., interleukin-6, IL-6) and type I interferons (IFN). Each TLR recognizes only specific PAMPs [47,51].

Today, 13 TLR family members have been identified in humans and mice together: TLR1–TLR10 in humans and TLR1–TLR9, TLR11–TLR13 in mice. TLR 1, 2, 4, 5, 6, and 10 are surface receptors and recognize PAMPs of bacteria, fungi, and protozoa, whereas TLR 3, 7, 8, 9, 11, 12, and 13 are only expressed in endocytic compartments [47]. Cell surface TLRs mainly respond to components of the microbial membrane such as proteins, lipids, and lipoproteins. Intracellular TLRs recognize non-self bacterial and viral nucleic acids, and also self-nucleic acids in autoimmunity [52].

**Figure 1:**
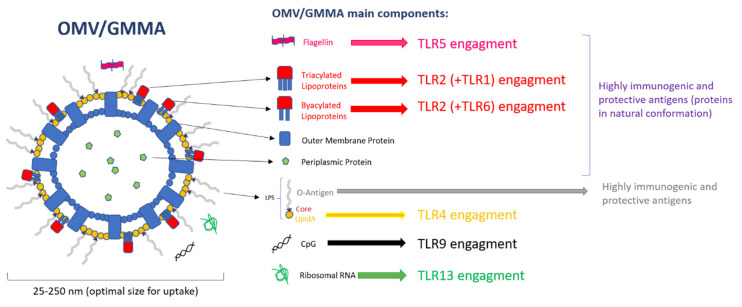
Outer Membrane Vesicle (OMV) components and Toll-like receptor (TLR) engagement.

OMVs faithfully resemble the composition of the bacterial outer membrane, and therefore contain LPS, glycerophospholipids, outer membrane proteins, lipoproteins, and soluble periplasmic proteins in the lumen; other molecules can be also present, mostly in low quantities and like impurities, such as flagellin in the case of OMVs produced from flagellated bacteria, ribosomal RNA, or double strand DNA. Therefore, TLR1, TLR2, TLR4, TLR5, TLR6, TLR9, and TLR13 are of major interest in the recognition of OMV PAMPs (Figure 1). Among them, the most important TLR for the recognition of PAMPs expressed on OMVs is TLR4, which is involved in the detection of the lipidA part of LPS [53] by forming a complex with the accessory proteins LPS Binding Protein (LBP), Myeloid Differentiation 2 (MD2), and CD14. Of particular interest are also TLR2, directly involved in the recognition of bacterial lipoproteins through dimerization with TLR1 in the case of tri-acylated lipoproteins recognition, or TLR6 in the case of binding of biacylated lipoproteins [54]. TLR5 is the receptor that recognizes flagellin, which may be present in OMVs preparations from flagellated bacteria, likely as FliC monomers [55,56]. TLR9, involved in recognition of unmethylated CpG motifs which are prevalent in bacterial but not vertebrate genomic DNAs [57], and TLR13, that recognizes bacterial ribosomal RNA [58], have a minor role in the recognition of OMV PAMPs, since these molecules are rarely found in OMV preparations.

On top of these receptors, all the other TLRs can be involved in the response to OMV stimulation, as OMVs can be efficiently decorated with homologous or heterologous antigens [59,60]. In particular, TLR3 (which recognizes dsRNA) and TLR7 (which senses ssRNA) can be involved in OMVs decoration with viral antigens [61], or TLR11/12 [62] in OMVs decoration with parasite antigens.

TLRs act in the form of dimers, and can be homo- (TLR 3, 4, 5, 7, 8, and 9) or hetero-dimers (TLR1, 2, and 6). PAMPs bind to TLRs as single molecules (lipoproteins that bind to TLR1-2 or TLR2-6 complexes [63]) or two molecules, as in the case of TLR4 homodimers that recognize two LPS molecules [64]. The affinity of the specific interaction between each TLR and its ligand starts from nanomolar concentrations of PAMPs, as in the case of TLR2, 3, 5, and 9 [60,65,66], or from higher ligand concentration, as for TLR4 [64], and it is different in different cells.

## 3. PAMPs on OMVs

LPS is typically considered the most potent and immune-stimulating component of OMVs. Its presence has attracted the attention of scientists developing OMV-based vaccines for two completely opposite point of views. On one side, the presence of LPS has raised safety concerns, due to its risk of inducing systemic reactogenicity when administered to humans (hence why LPS have been historically called endotoxins). On the other side, the presence of LPS provides major support to OMVs’ ability to induce an optimal immune stimulation compared to subunit vaccines. Fine tuning the balance between the risk of reactogenicity and the ability to efficiently stimulate the immune response is the key in OMV-based vaccine development [39].

The lipid A part of the LPS is the portion directly interacting with TLR4, and changes in its composition markedly affect its binding to and recognition by TLR4, resulting in low agonist or antagonist behavior, and with a great influence on the dimerization and downstream signaling of TLR4/MD-2.

**Figure 2:**
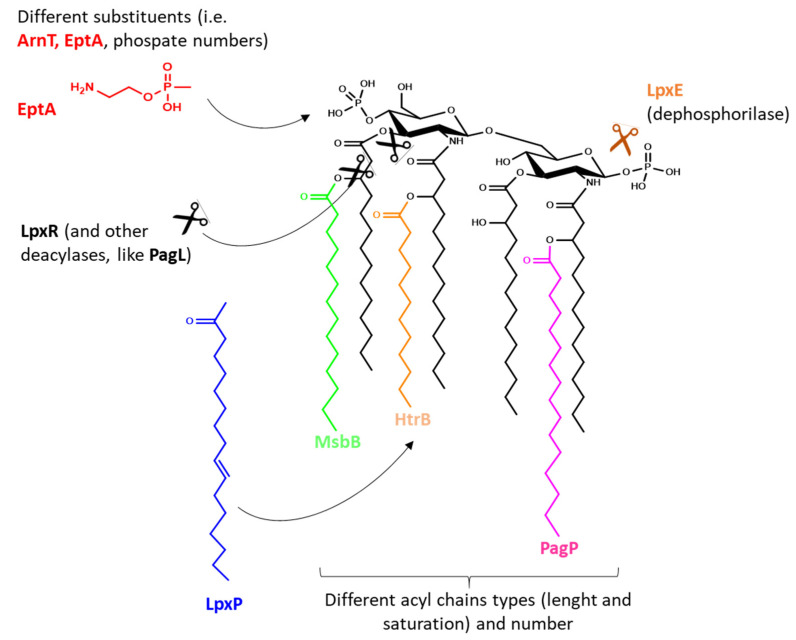
Examples of enzymes responsible for lipid A modifications used by bacteria to evade the innate immune recognition. Same mutations can be used to reduce an overly strong TLR4 stimulation from OMV-based vaccines and identify an optimal balance between safety and immunogenicity.

In most Gram-negative bacteria (i.e., *E. coli, Shigella*, *Salmonella*), the basic lipid A structure is composed of a β-1′,6-linked disaccharide of glucosamine phosphorylated at the 1 and 4′ positions and acylated at 2, 3, 2′ and 3′ position with R-3-hydroxymyristate (called lipid IV A). Several enzymes may act to decorate this scaffold in various positions, especially with fatty acids differing in length and saturation level, or by adding (or deleting) substituents to phosphoryl groups of glucosamines (Figure 2) [67]. In nature changes in the basic structure of lipid A are extensively used by bacteria in response to various shocks and to modulate host–pathogen interaction, and thus evade the innate immune recognition [67,68]. In *E. coli* and *Shigella*, the late acyltransferases HtrB [69] (also called LpxL) and MsbB [70] (also called LpxM) transfer a lauroyl fatty acid and a myristoyl fatty acid to the 3′ or 2′ position, respectively, resulting in the most reactogenic form of lipid A (an hexa-acylated glucosamine disaccharide phosphorylated at the 1 and 4′ position with acyl chains from 12 to 14 carbons in length and an asymmetric (4/2) distribution). The addition of a seventh fatty acid chain to the hexa-acylated lipid A by a 2-O-palmitoylation can be catalyzed by PagP [71], while deacylation by LpxR [72] or PagL [73] (Figure 2). The addition of phosphatidylethanolamine or arabinose to 1 and 4′ phosphate groups by EptA and ArnT [74], the deletion of a phosphoryl group by LpxE, or the hydroxylation by LpxO represent other widely described examples of enzymes acting on different lipid A sites [75,76]. Furthermore, different enzymes can act on the same position of the lipid A molecule, catalyzing the substitution of some acyl chains with others (i.e., LpxP can add a palmitoleoyl chain in the same site in which HtrB acts [77]), and many enzymes have different substrates specificity depending on the conditions (i.e., attachment of acyl chains differing for 2–4 carbons in length depending on stress conditions or metabolites present). Thus, a broad range of lipidA types are described and possible, all providing to the bacteria, and therefore to the OMVs, a different ability to activate TLR4, with the different structures possessing different levels of agonist or antagonist abilities [78].

In the case of the recognition of LPS, the accessory protein LBP forms direct contact with the lipidA of OMV and allows the extraction of an LPS molecule mediated by CD14 [79]. The complex LPS/LBP/CD14 interacts stably with TLR4, which becomes a functional LPS receptor after dimerization [80]. The LPS molecule is then transferred via this complex to MD2, which ultimately leads to the production of proinflammatory cytokines and chemokines, hence creating the key players in innate immunity and the modulators of adaptive immune responses [8]. Lipid A molecules with less than six acyl chains have a minimal potential to crosslink more than one TLR4-MD2 heterodimer [64], and therefore cause much weaker inflammatory activity compared to more acylated lipidA. Therefore, the reduction of lipidA chain number represents a strategy widely used to reduce an overly strong TLR4 stimulation, which is necessary for OMV use in humans.

Given that most of the genes involved in the lipid A biosynthesis are essential to maintain the integrity of the bacterial membrane and the vitality of the bacterium, successful approaches to modify the acylation status of the lipid A generating minimal defects to bacterial growth have been focused on the inactivation of the genes encoding the late acyltransferases, alone or in combination. For example, the deletion of genes encoding HtrB (*Shigella* [31,39,81]), or MsbB (in *Vibrio* [82]), or MsbB PagP (in *Salmonella* [35]) resulted in the production of OMVs carrying predominantly penta-acylated lipidA, which cause a markedly lower pro-inflammatory cytokine release compared to their respective OMVs carrying wild-type lipid A. Other modifications of lipid A tested preclinically consisted of the dephosphorylation of *Salmonella enterica* lipid A by knocking-in the dephosphorylases encoded by *lpxE* from *Francisella tularensis*, or by overexpressing the deacylases encoded by *lpxR*, resulting in the cleavage of two fatty acid chains, or PagL, which is a 3-O-deacylases. The reduction of lipid A endotoxic activity has also been essential for the development of meningococcal OMV-based vaccines. One way to reduce the OMV’s LPS content is by detergent extraction (e.g., used in Bexsero) [27]. An alternative method consists of the generation of meningococcal strains with genetically detoxified LPS, in particular knock out of *lpxL1* [38,83] or *lpxL2* [84] genes, both resulting in penta-acylated lipid A structures, which results in a strongly attenuated endotoxic activity, with tolerability demonstrated both preclinically and clinically.

OMVs have also been shown to contain other PAMPs in addition to LPS (Figure 1). For instance, *Shigella* [81], *Salmonella* [35], and *Neisseria meningitidis* [38] GMMA have all been demonstrated to efficiently activate TLR2 using Human Embryonic Kidney cells stably transfected to express just specific TLRs, in line with their content of lipoproteins in natural conformation. Also, target blocking TLRs in human PBMC, alone or in combination, has elucidated the relative contribution of TLR2, TLR4, and TLR5 in the residual ability to induce pro-inflammatory response of *Shigella* [81] and *Salmonella* [35] GMMA with modified lipidA. These experiments demonstrated that TLR4 contribution is drastically reduced to a level in which pro-inflammatory response is mostly mediated by TLR2 activation, and the relative contribution of PRRs other than TLR2, TLR4, and TLR5 is marginal (<10%) in the residual response. Studies utilizing LPS-deficient OMVs from *Neisseria* demonstrated the ability of non-LPS PAMPs to enhance immune responses to vesicles [85,86]. Although presence of flagellin and CpG DNA has been detected in *Salmonella* GMMA [35] and *P. aeruginosa* vesicles [24,87], to date no studies have demonstrated that they directly impact the host responses to intact vesicles containing LPS. The same is true for the low presence of cytoplasmic proteins, principally ribosomal proteins, detected in *Shigella* GMMA preparations, likely purified together with GMMA [88].

## 4. Engagement of Innate and Adaptive Immune Response Induced upon Activation by PAMPs

TLRs have been recognized to module host innate and adaptive immune responses through the activation of immune cell signaling.

The maturation of DCs is key for the initiation of antigen-specific immune responses [89]. The upregulation of co-stimulatory molecules, such as CD80 and CD86, and the modulation of the peptide-MHC (major histocompatibility complex) on DCs following TLR stimulation allows DCs to prime naïve T cells [90,91]. Indeed, TLR agonists, particularly LPS, facilitate the redistribution of MHC class I and II molecules to the surface of DCs [92]. Moreover, TLR signaling drives endosomes acidification in DCs, promoting peptide loading onto MHC molecules [93,94] and promoting cross presentation of foreign antigens for CD8^+^ T cell response stimulation by helping the fusion of MHC-I-containing endosomes with phagosomes [93,95]. The migration of DCs from the periphery to the draining lymph node, where naïve T cell stimulation may take place, is stimulated by PRR activation.

When DCs recognize a microbial product, different effector responses may occur, determined by the receptors that are stimulated and the cytokines that are released from the DCs. Therefore, a naïve T cell can differentiate into Th1-, Th2- or Th17 T cell, depending on the type of antigen presenting cell involved [96]. Usually, signaling via TLR3, TLR4, TLR7, TLR8, and TLR9 induces Th1-type immune responses, while signaling via TLR2 (together with TLR1 or TLR6) and TLR5 promotes Th2-type immune responses [90,91]. For instance, in response to *H. pylori* and *P. aeruginosa* OMVs, epithelial cells produce IL-8, a cytokine involved in neutrophil and monocyte recruitment in vivo [87,97]. Instead, OMVs from *Salmonella* Typhimurium induce an increase of MHC-II and CD86 surface expression on macrophages and DCs and an enhanced production of nitric oxide, tumor necrosis factor-α, and IL-12. Which TLR is activated by the exogenous stimulus obviously depends on which DCs are activated. Indeed, DCs are functionally heterogeneous and have been classified in different subsets that express subset-specific PRRs [98,99,100]. As a result, these cells are activated in response to different stimuli, which in turn trigger specific signaling pathways leading to the production of specific cytokines that designate the Th cell subset that will be developed [101]. For example, plasmacytoid DCs induce Type I IFNs and thus control anti-viral immunity [98,99,100]. They do not express surface TLRs to detect bacterial PAMPs but only present endosomal TLRs, such as TLR7 and 9, that recognize ssRNA and CpG DNA, respectively. CD8a^+^ DCs, a different DC subset which drives cross-presentation, is relevant for the activation of CD8^+^ T cells. These DCs present TLR3 as well as RLRs, recognize viral antigens, and produce Type I IFNs and IL-12 [102] (Table 1).

TLRs are also expressed by T cells. For instance, TLR ligands influence Treg development [115], either directly, since they are expressed on this T cell subset, or indirectly, when a Treg interacts with TLR-activated antigen presenting cells [116]. Treg cell activation or suppression, upon the stimulation of the TLR signaling pathway, is controlled by the type of TLR agonist and its ability to elicit antagonistic Th17 cell differentiation [115]. This feature is important, especially for diseases where Th17-type cytokines (IL-17A, IL-17F and IL-22) [115,117] have a prominent role, such as autoimmunity, chronic inflammation, and cancer.

In B cells, TLR signaling enhances antigen uptake through the upregulation of MHC molecules and cross-talk with T cells through the expression of costimulatory molecules, thus promoting the secretion of antigen-specific antibodies upon vaccination with antigens in combination with TLR ligands [118].

B cell responses can be divided into T cell-independent and T cell-dependent. T-cell-independent B cell responses are driven by innate-like B cells which express TLRs and respond to microbiota derived stimuli. In these cells, NF-kB signaling is induced upon the activation of both TLRs and B cell receptors and leads to the production of natural immunoglobulin (IgM) which protect against bacteria and viruses such as influenza [119]. T cell-dependent antibody responses are initiated by the engagement of the B cell receptor by the antigen presented on an antigen presenting cell and are modulated by cytokines that drive isotype class switching. Antibody responses, especially IgG2 in mice or IgG1 or IgG3 in humans, are determined by TLRs present on B cells (Table 1) [120].

In the last few years, several synthetic TLR agonists (reviewed in [121]) have been selected as molecules that can be used as vaccine adjuvants and have been tested in clinical trials. One of them is the TLR4 agonist monophosphoryl lipid A (MPL), a detoxified form of the bacterial LPS. MPL formulated with aluminum hydroxide, named AS04, has been approved for use in vaccines that target human papilloma virus and hepatitis B [122]. It has been demonstrated to bind TLR4, thus promoting cytokine production, antigen presentation, and the migration of antigen presenting cells to the T cell area of draining lymph nodes, where they can prime naïve T cells [122]. Non-methylated CpG oligonucleotide has also been identified as a novel adjuvant and has been used in both preclinical and clinical studies for cancer vaccines [123]. It stimulates TLR9 expressed by human plasmacytoid DCs and B cells, thus promoting B- and T-cell responses. Other TLR agonists that have demonstrated promising pre-clinical and clinical results as vaccine adjuvants are those targeting the TLR7/8 pathway. Two small molecules, i.e., Imiquimod, an agonist of TLR7, and Resiquimod, a TLR7/8 agonist, have been conjugated to protein antigens or properly formulated and shown an improvement in the immunogenicity of the tested vaccine [123,124,125]. Imiquimod has been proven to modulate the immune response when used as a topic vaccine adjuvant. Indeed, when immature DCs were injected into Imiquimod-pretreated skin of cancer patients, they migrated to draining lymph nodes and showed enhanced DC immunostimulatory capacity [123]. Imiquimod has been licensed as a topical treatment for malignant and non-malignant skin cell disorders and has been demonstrated to be safe and to have a good efficacy [126,127,128,129,130].

OMVs possess the ability to stimulate the innate compartment of a host’s immune system through the activation of TLRs and NLRs since they contain various bacterial PAMPs such as LPS, lipoproteins, flagellin monomers, and bacterial DNA fragments [8]. In recent studies, the intramuscular injection of OMVs with heterologous antigens enhanced antigen-specific humoral and cellular immune responses and increased the protection rate against tumor and virus challenges [131,132]. OMVs with attenuated endotoxicity (fmOMVs) through the modification of the lipid A moiety of LPS, have been tested as a mucosal adjuvant using an influenza vaccine model. fmOMVs have been demonstrated to be less reactogenic when compared with native OMVs (nOMVs), and intranasal injection of vaccine antigens with fmOMVs resulted in the enhanced production of IgG and IgA compared with the influenza vaccine adjuvanted with cholera toxin. Furthermore, co-administration of fmOMVs conferred protection against homologous and heterologous viral challenges, indicating that fmOMVs can be a promising mucosal adjuvant for intranasal vaccines [133].

The capacity of OMVs to trigger an adaptive memory immune response has already been demonstrated with several OMV-based acellular vaccines, the most successful example being represented by vaccines against *N. meningitidis* serogroup B. The advancement and characteristics of such vaccines have recently been comprehensively reviewed by Holst and colleagues [134]. As described in this work, OMVs have been confirmed to be the only effective formulation against serogroup B infections, with over 55 million doses administered to date. Several approaches to vaccine formulation have been adopted, targeting different strains and including antigens specific to a given geographic region. All preparations of meningococcal vesicles have been proven to elicit a protective bactericidal humoral response mainly targeting the outer membrane porins PorA and PorB [135]. The adjuvant role of LPS in OMVs seems to be important for the induction of an effective immune response. In fact, immune responses to LPS-free *N. meningitidis* OMVs are poor, and need the inclusion of exogenous PAMPs as immune stimulators in order to generate an optimal immune response [85,86,136]. The most commonly used commercial OMV-based vaccine against *N. meningitidis* serogroup B is based on detergent-extracted vesicles. This OMV purification process markedly decreases the endotoxic content of vesicles. Recent studies investigating the mechanism underlying the impact of TLR activation on the maturation of phagosomes and DCs and on the antigen presentation on MHC molecules, demonstrated that a “mixture” of LPS and other outer membrane bacterial PAMPs could generate a more effective response [93]. Schild et al. demonstrated that OMVs from *Vibrio cholerae* generate a protective antibody response in a neonatal mouse model of passive antibody transfer [137]. B-cell responses to OMVs from *S*. Typhimurium [138], *B. burgdorferi* [139], and *Flavobacterium* [140] have also been reported, suggesting that OMVs can be good antigen delivery systems to generate effective antibody responses.

## 5. OMVs Compared to Classical Vaccines

The intrinsic features of OMVs have been exploited in the vaccinology field over the past few decades (Table 2) and their potential as novel vaccine delivery platform has been investigated by several groups. For instance, when *N. meningitidis* OMVs displaying the antigenic porin protein PorA were examined in comparison to purified PorA protein, a humoral immune response was elicited in response to both candidate vaccines, but only the PorA-containing OMVs induced bactericidal antibodies [141]. The functional immune response induced by OMVs over-expressing fHbp is similar to that induced by > 10-fold higher fHbp administered in recombinant form, suggesting that the native conformation and self-adjuvant properties of OMV have a great impact in the immune response induced [142]. OMVs naturally released from a recombinant African *N. meningitidis* W strain with deleted capsule locus, attenuated LPS toxicity (*lpxL1* mutant), improved OMV yield (*gna33* mutant) and with overexpressed fHbp v.1 have been proposed as an affordable vaccine with broad coverage against strains from all main serogroups currently causing meningococcal meningitis in sub-Saharan Africa [38]. Recently, novel *B. pertussis* OMV vaccines have been compared to the current approved whole cell *B. pertussis* vaccine, and proved to efficiently raise OMV-specific antibodies in mice to a level comparable to that obtained with the whole cell vaccine [28,143,144]. Moreover, OMVs were more effective against a current circulating isolate than the whole-cell vaccine and able to induce long-lasting immunity [144]. Also GMMA from *S.* Typhimurium and *S.* Enteritidis strains engineered to increase OMV yields induced high anti-O polysaccharides specific IgG titers comparable to those induced by corresponding CRM197 glycoconjugates formulated with alhydrogel, and with increased IgG antibody isotype profile and complement-mediated bactericidal activity [34].

More recently OMVs have been explored as delivery systems of specific proteins or polysaccharides, as they can be easily decorated with heterologous antigens. OMVs, expressing the poorly immunogenic green-fluorescent protein (GFP), elicited strong and sustained anti-GFP antibody titers in immunized mice, whereas immunization with GFP alone did not elicit such titers [150]. Mice immunized intranasally with *S.* Typhimurium OMVs, engineered to contain the pneumococcal protein PspA in the lumen, developed serum antibody responses against PspA, *Salmonella* LPS, and outer membrane proteins, while no detectable responses were developed in mice immunized with an equivalent dose of recombinant PspA [145]. Immunization with 50 μg of *E. coli* OMVs expressing 0.1 μg Omp22 of *A. baumannii* rapidly generated significantly higher Omp22-specific antibodies than immunization with 50 μg recombinant protein formulated with Alum, while injection with 0.5 μg recombinant Omp22 with Alum produced no detectable response [146]. Other examples have been reported, demonstrating that OMVs often induce humoral immune responses far more efficiently than recombinant antigenic proteins formulated with alum [38,148,149].

Recently, *E. coli* OMVs have been used as carriers for the expression of heterologous polysaccharides, resulting in glycoengineered OMVs (glyOMVs) [151]. Using this approach, Chen et al. [147] created glyOMVs displaying O-polysaccharides from *F. tularensis*. In mice, immunization with glyOMVs led to a sustained O-polysaccharide-specific IgG production which was 2–3-fold higher than IgG levels elicited by the native LPS and protected the animals against lethal challenge with *F. tularensis*. Upon subcutaneous glyOMV administration, a protective mucosal immune response was also generated. Price et al. [152] engineered *E. coli* OMVs to express capsular polysaccharides, such as a *S. pneumoniae* CPS14 capsule. The corresponding glyOMVs induced IgG levels and efficacy in opsonophagocytic activity tests comparable to those induced by PCV13.

Multiple factors can explain the strong immune response induced by OMVs. In fact, not only OMVs contain TLR agonists providing self-adjuvanticity, but antigens are also presented in multiplicity in the context of a membrane and in their native orientation and conformation, as well as OMVs have the optimal size for immune stimulation [30,59]. To our knowledge, few studies have been performed so far to elucidate the specific contribution of TLR activation to the immune response induced by OMVs. The roles of TLR2 and TLR4 have been investigated in the induction of immune responses in mice after immunization with *N. meningitidis* OMVs [35,153]. Wild type mice were compared with mice deficient in TLR2, TLR4, or TRIF and their innate and adaptive immune responses were analyzed. TRIF-deficient and TLR4-deficient mice showed reduced immune response after immunization, whereas it was not impaired in TLR2-deficient mice, suggesting that TLR4 (but not TLR2) stimulation contributes to the immunogenicity of the *N. meningitidis* OMVs.

Like OMVs, Outer Membrane Protein Complexes (OMPCs) have been proposed as particle platforms for antigen display and also possess inherent adjuvant activity. It has been shown that chemical conjugation of malaria transmission blocking antigens Pfs25 or Pfs230 to OMPCs enhanced the immunogenicity and functional activity of the antigens compared to Pfs25 or Pfs230 alone adjuvanted or conjugated to *r*EPA carrier protein [45,154]. OMPCs have been shown to possess TLR2-mediated adjuvant activity attributed to its membrane porin proteins [155]. It has been demonstrated that *Haemophilus influenzae* type b-OMPC glycoconjugate vaccine induces cytokine production by engaging human TLR2 and requires the presence of TLR2 for optimal immunogenicity. Furthermore, OMPC may contain TLR4 agonists such as LPS since they derive from the outer membrane of Gram-negative bacteria.

## 6. Discussion

OMVs combine both adjuvant and carrier activities, which allows for an increase in the low immunogenic properties of protein and carbohydrate antigens alone. The presence of TLR agonists on OMVs plays an unmistakably important role on their ability to efficiently stimulate both innate and adaptive immunity. However, if limited activation of the innate immune system can aid a useful immune response to the vaccine, a strong activation could lead to adverse effects ranging from febrile response to septic shock [156]. Thus, a balance between immune stimulation and reactogenicity is desired in a vaccine. Mass vaccination with detergent-extracted OMV-based vaccines are well tolerated and are currently changing the world by highly decreasing the burden of meningococcal meningitis [157]. Similarly, results obtained in terms of tolerability of GMMA are extremely satisfactory, and true not only in healthy adults in developed countries, but also in developing countries, where endemic factors may influence the immunogenicity/reactogenicity balance [158]. However, due to differences in the maturation of TLR in children and adults [159], an open question remains regarding the risk of systemic reactogenicity in different age groups. For instance, neonatal monocytes displayed down-regulated surface expression of CD14 and TLR4 and suppressed phosphorylation of NF-κB p65 and p38 MAPK in response to the TLR4 agonist LPS stimulation when compared with adult monocytes [87]. For vaccines targeting children, the systemic reactogenicity can be fully assessed only by ad hoc dose escalation clinical trials.

Overall, OMVs represent an attractive and innovative platform for the development of effective and safe vaccines. Nevertheless, few studies so far have investigated the OMV mode of action, including the role of TLR agonists on the induced immune response. Such studies will be important for driving the rational design of improved OMV-based vaccines. Moreover, important differences exist between mice and humans regarding function and tissue expression of TLRs. For example, TLR2, which has been shown to be the main driver of detoxified *Shigella* [81] and *Salmonella* [35] GMMA reactogenicity, presents differences between humans and mice, since human but not murine TLR2 discriminates between tri-palmitoylated and tri-lauroylated peptide [160]. The recognition of lipid A by the TLR4/MD-2 complex is species-specific [85] with mice being less sensitive to LPS than humans [64]. In addition, within human subjects, TLR4 presents polymorphisms that result in different susceptibility to LPS [86]. Both in mice and humans, TLR7 is expressed in B cells, neutrophils, and pDC; however, in mice TLR7 is expressed by macrophages and CD8^−^, but not CD8^+^ and DC subsets [116]. TLR8, in contrast, is expressed by monocytes lineage cells and myeloid DC in man, whereas it may not be a functional receptor in mice [161]. TLR9 has been recognized as the mediator of species-specific DNA sequence recognition [162,163]. All this implies that TLR species-specific properties exist and thus humans and mice may have distinct susceptibilities to challenge with specific TLR ligands. Thus, in parallel to additional investigation in animal models to dissect OMV mechanisms, clinical trials will be fundamental to better characterize OMV vaccine immunological properties in the target populations and to guarantee their efficacy and safety.

## Figures and Tables

**Table 1:** Expression of principal TLRs on different human immune cells.

TLR Type	Absent/Low Expression	Medium/High Expression
TLR2 (+TLR1)	plasmacytoid DCs, B cells * [103]	monocytes [104], myeloid DCs [105], T cells [106]
TLR2 (+TLR6)	monocytes [107], plasmacytoid DCs, B cells * [103]	myeloid DCs [105], T cells [106]
TLR3	monocytes [104], plasmacytoid DCs	CD8a^+^ myeloid DCs [105], CD138^+^ B cells [103], T cells [108]
TLR4	plasmacytoid DCs	monocytes [104], myeloid DCs [105], CD138^+^ B cells [103], T cells [109]
TLR5	plasmacytoid DCs, B cells	monocytes [107], myeloid DCs [105], B cells [110], T cells [111]
TLR7		monocytes [112], plasmacytoid DCs, myeloid DCs, B cells [103], T cells [113]
TLR8	plasmacytoid DCs	monocytes [112], myeloid DCs [105], B cells [103], T cells [113]
TLR9		monocytes [107], plasmacytoid DCs, B cells [103], T cells [114]

* Only activated and memory B cells, whereas naïve B cells express low levels of all TLRs.

**Table 2:** OMVs versus classical vaccines. Examples of animal studies in which OMVs have shown improved immunogenicity compared to traditional formulations.

OMVs	Classical Vaccines	
Purified PorA on OMV	Purified PorA	[141]
*Salmonella* Typhimurium OMV expressing PspA in the lumen	Recombinant PspA	[145]
*E. coli* OMVs expressing Omp22 of *A. baumannii*	Recombinant Omp22 adjuvanted with Alum	[146]
OMVs over-expressing fHbp	Recombinant fHbp	[38,142]
TdapOMVsBp	Whole cell *B. pertussis* vaccine and acellular vaccines	[28]
OMVs from *B. pertussis* B1917	Whole cell *B. pertussis* vaccine and acellular vaccines	[143]
*S*. Typhimurium and *S*. Enteritidis GMMA	*S*. Typhimurium and *S*. Enteritidis O-antigen conjugated to CRM197 and formulated on Alhydrogel	[34]
glyOMVs displaying O-polysaccharides from *F. tularensis*	Native *F. tularensis* LPS	[147]
*E. coli* OMV expressing *Chlamydia* rHtrA	Purified *Chlamydia* rHtrA	[148]
*E. coli* OMVs carrying different heterologous antigens in their lumen	Purified heterologous antigens	[149]

## Abbreviations

OMV	Outer Membrane Vesicle
LPS	Lipopolysaccharide
TLR	Toll Like Receptor
PAMP	Pathogen-Associated Molecular Pattern
PRR	Pattern Recognition Receptor
GMMA	Generalized Modules for Membrane Antigens
DAMP	Damage-Associated Molecular Pattern
RLR	RIG-I-Like Receptor
NLR	NOD-Like Receptor
TIR	Toll/interleukin-1 receptor
IL	interleukin
IFN	interferon
MHC	major histocompatibility complex
DC	dendritic cell
fmOMV	OMV with attenuated endotoxicity
nOMV	native Outer Membrane Vesicle
OMPC	Outer Membrane Protein Complex
LBP	LPS Binding Protein
MD2	Myeloid Differentation 2

## References

[1] Schwechheimer C., Kuehn M.J. (2015). Outer-membrane vesicles from Gram-negative bacteria: Biogenesis and functions. Nat. Rev. Microbiol..

[2] Wensink J., Witholt B. (1981). Conversion of Free Lipoprotein to the Murein-Bound Form. Eur. J. Biochem..

[3] Bohuszewicz O., Liu J., Low H.H. (2016). Membrane remodelling in bacteria. J. Struct. Biol..

[4] Roier S., Zingl F.G., Cakar F., Durakovic S., Kohl P., Eichmann T.O., Klug L., Gadermaier B., Weinzerl K., Prassl R. (2016). A novel mechanism for the biogenesis of outer membrane vesicles in Gram-negative bacteria. Nat. Commun..

[5] Sutterlin H.A., Shi H., May K.L., Miguel A., Khare S., Huang K.C., Silhavy T.J. (2016). Disruption of lipid homeostasis in the Gram-negative cell envelope activates a novel cell death pathway. Proc. Natl. Acad. Sci. USA.

[6] McBroom A.J., Kuehn M.J. (2007). Release of outer membrane vesicles by Gram-negative bacteria is a novel envelope stress response. Mol. Microbiol..

[7] Gerritzen M.J.H., Martens D.E., Uittenbogaard J.P., Wijffels R.H., Stork M. (2019). Sulfate depletion triggers overproduction of phospholipids and the release of outer membrane vesicles by *Neisseria meningitidis*. Sci. Rep..

[8] Kaparakis-Liaskos M., Ferrero R.L. (2015). Immune modulation by bacterial outer membrane vesicles. Nat. Rev. Immunol..

[9] Reidl J. (2016). Outer Membrane Vesicle Biosynthesis in Salmonella: Is There More to Gram-Negative Bacteria?. mBio.

[10] Elhenawy W., Bording-Jorgensen M., Valguarnera E., Haurat M.F., Wine E., Feldman M.F. (2016). LPS Remodeling Triggers Formation of Outer Membrane Vesicles in Salmonella. mBio.

[11] Athman J.J., Wang Y., McDonald D.J., Boom W.H., Harding C.V., Wearsch P.A. (2015). Bacterial Membrane Vesicles Mediate the Release of *Mycobacterium tuberculosis* Lipoglycans and Lipoproteins from Infected Macrophages. J. Immunol..

[12] Mashburn-Warren L.M., Whiteley M. (2006). Special delivery: Vesicle trafficking in prokaryotes. Mol. Microbiol..

[13] Lee E.Y., Choi D.S., Kim K.P., Gho Y.S. (2008). Proteomics in gram-negative bacterial outer membrane vesicles. Mass Spectrom. Rev..

[14] Bonnington K.E., Kuehn M.J. (2014). Protein selection and export via outer membrane vesicles. Biochim. Biophys. Acta.

[15] Unal C.M., Schaar V., Riesbeck K. (2011). Bacterial outer membrane vesicles in disease and preventive medicine. Semin. Immunopathol..

[16] Horstman A.L., Kuehn M.J. (2000). Enterotoxigenic *Escherichia coli* secretes active heat-labile enterotoxin via outer membrane vesicles. J. Biol. Chem..

[17] Chatterjee D., Chaudhuri K. (2011). Association of cholera toxin with *Vibrio cholerae* outer membrane vesicles which are internalized by human intestinal epithelial cells. FEBS Lett..

[18] Lindmark B., Rompikuntal P.K., Vaitkevicius K., Song T., Mizunoe Y., Uhlin B.E., Guerry P., Wai S.N. (2009). Outer membrane vesicle-mediated release of cytolethal distending toxin (CDT) from *Campylobacter jejuni*. BMC Microbiol..

[19] Pettit R.K., Judd R.C. (1992). Characterization of naturally elaborated blebs from serum-susceptible and serum-resistant strains of *Neisseria gonorrhoeae*. Mol. Microbiol..

[20] Schooling S.R., Beveridge T.J. (2006). Membrane Vesicles: An Overlooked Component of the Matrices of Biofilms. J. Bacteriol..

[21] Palsdottir H., Remis J.P., Schaudinn C., O‘Toole E., Lux R., Shi W., McDonald K.L., Costerton J.W., Auer M. (2009). Three-dimensional macromolecular organization of cryofixed *Myxococcus xanthus* biofilms as revealed by electron microscopic tomography. J. Bacteriol..

[22] Kadurugamuwa J.L., Beveridge T.J. (1995). Virulence factors are released from *Pseudomonas aeruginosa* in association with membrane vesicles during normal growth and exposure to gentamicin: A novel mechanism of enzyme secretion. J. Bacteriol..

[23] Fulsundar S., Harms K., Flaten G.E., Johnsen P.J., Chopade B.A., Nielsen K.M. (2014). Gene transfer potential of outer membrane vesicles of *Acinetobacter baylyi* and effects of stress on vesiculation. Appl. Environ. Microbiol..

[24] Renelli M., Matias V., Lo R.Y., Beveridge T.J. (2004). DNA-containing membrane vesicles of *Pseudomonas aeruginosa* PAO1 and their genetic transformation potential. Microbiology.

[25] Kuehn M.J., Kesty N.C. (2005). Bacterial outer membrane vesicles and the host-pathogen interaction. Genes Dev..

[26] Beveridge T.J. (1999). Structures of gram-negative cell walls and their derived membrane vesicles. J. Bacteriol..

[27] Dowling D.J., Sanders H., Cheng W.K., Joshi S., Brightman S., Bergelson I., Pietrasanta C., van Haren S.D., van Amsterdam S., Fernandez J. (2016). A Meningococcal Outer Membrane Vesicle Vaccine Incorporating Genetically Attenuated Endotoxin Dissociates Inflammation from Immunogenicity. Front. Immunol..

[28] Bottero D., Gaillard M.E., Zurita E., Moreno G., Martinez D.S., Bartel E., Bravo S., Carriquiriborde F., Errea A., Castuma C. (2016). Characterization of the immune response induced by pertussis OMVs-based vaccine. Vaccine.

[29] Pritsch M., Ben-Khaled N., Chaloupka M., Kobold S., Berens-Riha N., Peter A., Liegl G., Schubert S., Hoelscher M., Löscher T. (2016). Comparison of Intranasal Outer Membrane Vesicles with Cholera Toxin and Injected MF59C.1 as Adjuvants for Malaria Transmission Blocking Antigens AnAPN1 and Pfs48/45. J. Immunol. Res..

[30] Tan K., Li R., Huang X., Liu Q. (2018). Outer Membrane Vesicles: Current Status and Future Direction of These Novel Vaccine Adjuvants. Front. Microbiol..

[31] Berlanda Scorza F., Colucci A.M., Maggiore L., Sanzone S., Rossi O., Ferlenghi I., Pesce I., Caboni M., Norais N., Di Cioccio V. (2012). High yield production process for Shigella outer membrane particles. PLoS ONE.

[32] McMahon K.J., Castelli M.E., García Vescovi E., Feldman M.F. (2012). Biogenesis of outer membrane vesicles in *Serratia marcescens* is thermoregulated and can be induced by activation of the Rcs phosphorelay system. J. Bacteriol..

[33] Kulp A.J., Sun B., Ai T., Manning A.J., Orench-Rivera N., Schmid A.K., Kuehn M.J. (2015). Genome-Wide Assessment of Outer Membrane Vesicle Production in *Escherichia coli*. PLoS ONE.

[34] Micoli F., Rondini S., Alfini R., Lanzilao L., Necchi F., Negrea A., Rossi O., Brandt C., Clare S., Mastroeni P. (2018). Comparative immunogenicity and efficacy of equivalent outer membrane vesicle and glycoconjugate vaccines against nontyphoidal Salmonella. Proc. Natl. Acad. Sci. USA.

[35] Rossi O., Caboni M., Negrea A., Necchi F., Alfini R., Micoli F., Saul A., MacLennan C.A., Rondini S., Gerke C. (2016). Toll-Like Receptor Activation by Generalized Modules for Membrane Antigens from Lipid A Mutants of *Salmonella enterica* Serovars Typhimurium and Enteritidis. Clin. Vaccine Immunol..

[36] De Benedetto G., Alfini R., Cescutti P., Caboni M., Lanzilao L., Necchi F., Saul A., MacLennan C.A., Rondini S., Micoli F. (2017). Characterization of O-antigen delivered by Generalized Modules for Membrane Antigens (GMMA) vaccine candidates against nontyphoidal Salmonella. Vaccine.

[37] Schager A.E., Dominguez-Medina C.C., Necchi F., Micoli F., Goh Y.S., Goodall M., Flores-Langarica A., Bobat S., Cook C.N.L., Arcuri M. (2018). IgG Responses to Porins and Lipopolysaccharide within an Outer Membrane-Based Vaccine against Nontyphoidal Salmonella Develop at Discordant Rates. mBio.

[38] Koeberling O., Ispasanie E., Hauser J., Rossi O., Pluschke G., Caugant D.A., Saul A., MacLennan C.A. (2014). A broadly-protective vaccine against meningococcal disease in sub-Saharan Africa based on generalized modules for membrane antigens (GMMA). Vaccine.

[39] Gerke C., Colucci A.M., Giannelli C., Sanzone S., Vitali C.G., Sollai L., Rossi O., Martin L.B., Auerbach J., Di Cioccio V. (2015). Production of a Shigella sonnei Vaccine Based on Generalized Modules for Membrane Antigens (GMMA), 1790GAHB. PLoS ONE.

[40] Launay O., Lewis D.J.M., Anemona A., Loulergue P., Leahy J., Sciré A.S., Maugard A., Marchetti E., Zancan S., Huo Z. (2017). Safety Profile and Immunologic Responses of a Novel Vaccine Against *Shigella sonnei* Administered Intramuscularly, Intradermally and Intranasally: Results From Two Parallel Randomized Phase 1 Clinical Studies in Healthy Adult Volunteers in Europe. EBioMedicine.

[41] Launay O., Ndiaye A.G.W., Conti V., Loulergue P., Sciré A.S., Landre A.M., Ferruzzi P., Nedjaai N., Schütte L.D., Auerbach J. (2019). Booster Vaccination With GVGH *Shigella sonnei* 1790GAHB GMMA Vaccine Compared to Single Vaccination in Unvaccinated Healthy European Adults: Results From a Phase 1 Clinical Trial. Front. Immunol..

[42] Van de Waterbeemd B., Streefland M., van der Ley P., Zomer B., van Dijken H., Martens D., Wijffels R., van der Pol L. (2010). Improved OMV vaccine against *Neisseria meningitidis* using genetically engineered strains and a detergent-free purification process. Vaccine.

[43] Koeberling O., Giuntini S., Seubert A., Granoff D.M. (2009). Meningococcal outer membrane vesicle vaccines derived from mutant strains engineered to express factor H binding proteins from antigenic variant groups 1 and 2. Clin. Vaccine Immunol..

[44] Watkins H.C., Rappazzo C.G., Higgins J.S., Sun X., Brock N., Chau A., Misra A., Cannizzo J.P.B., King M.R., Maines T.R. (2017). Safe Recombinant Outer Membrane Vesicles that Display M2e Elicit Heterologous Influenza Protection. Mol. Ther..

[45] Scaria P.V., Rowe C.G., Chen B.B., Muratova O.V., Fischer E.R., Barnafo E.K., Anderson C.F., Zaidi I.U., Lambert L.E., Lucas B.J. (2019). Outer membrane protein complex as a carrier for malaria transmission blocking antigen Pfs230. NPJ Vaccines.

[46] Grandi A., Tomasi M., Zanella I., Ganfini L., Caproni E., Fantappiè L., Irene C., Frattini L., Isaac S.J., König E. (2017). Synergistic Protective Activity of Tumor-Specific Epitopes Engineered in Bacterial Outer Membrane Vesicles. Front. Oncol..

[47] Kawai T., Akira S. (2010). The role of pattern-recognition receptors in innate immunity: Update on Toll-like receptors. Nat. Immunol..

[48] Kumar H., Kawai T., Akira S. (2009). Toll-like receptors and innate immunity. Biochem. Biophys. Res. Commun..

[49] Anderson K.V., Nusslein-Volhard C. (1984). Information for the dorsal-ventral pattern of the Drosophila embryo is stored as maternal mRNA. Nature.

[50] Jin M.S., Lee J.O. (2008). Structures of TLR-ligand complexes. Curr. Opin. Immunol..

[51] Mahla R.S., Reddy M.C., Prasad D.V.R., Kumar H. (2013). Sweeten PAMPs: Role of Sugar Complexed PAMPs in Innate Immunity and Vaccine Biology. Front. Immunol..

[52] Blasius A.L., Beutler B. (2010). Intracellular toll-like receptors. Immunity.

[53] Poltorak A., He X., Smirnova I., Liu M.Y., Van Huffel C., Du X., Birdwell D., Alejos E., Silva M., Galanos C. (1998). Defective LPS signaling in C3H/HeJ and C57BL/10ScCr mice: Mutations in Tlr4 gene. Science.

[54] Kang J.Y., Nan X., Jin M.S., Youn S.J., Ryu Y.H., Mah S., Han S.H., Lee H., Paik S.G., Lee J.O. (2009). Recognition of lipopeptide patterns by Toll-like receptor 2-Toll-like receptor 6 heterodimer. Immunity.

[55] Gewirtz A.T., Navas T.A., Lyons S., Godowski P.J., Madara J.L. (2001). Cutting edge: Bacterial flagellin activates basolaterally expressed TLR5 to induce epithelial proinflammatory gene expression. J. Immunol..

[56] Hayashi F., Smith K.D., Ozinsky A., Hawn T.R., Yi E.C., Goodlett D.R., Eng J.K., Akira S., Underhill D.M., Aderem A. (2001). The innate immune response to bacterial flagellin is mediated by Toll-like receptor 5. Nature.

[57] Hemmi H., Takeuchi O., Kawai T., Kaisho T., Sato S., Sanjo H., Matsumoto M., Hoshino K., Wagner H., Takeda K. (2000). A Toll-like receptor recognizes bacterial DNA. Nature.

[58] Hidmark A., von Saint Paul A., Dalpke A.H. (2012). Cutting edge: TLR13 is a receptor for bacterial RNA. J. Immunol..

[59] Gerritzen M.J.H., Martens D.E., Wijffels R.H., van der Pol L., Stork M. (2017). Bioengineering bacterial outer membrane vesicles as vaccine platform. Biotechnol. Adv..

[60] Olguín Y., Villalobos P., Carrascosa L.G., Young M., Valdez E., Lechuga L., Galindo R. (2013). Detection of flagellin by interaction with human recombinant TLR5 immobilized in liposomes. Anal. Bioanal. Chem..

[61] Fitzgerald K.A., Kagan J.C. (2020). Toll-like Receptors and the Control of Immunity. Cell.

[62] Yarovinsky F., Zhang D., Andersen J.F., Bannenberg G.L., Serhan C.N., Hayden M.S., Hieny S., Sutterwala F.S., Flavell R.A., Ghosh S. (2005). TLR11 activation of dendritic cells by a protozoan profilin-like protein. Science.

[63] Jin M.S., Kim S.E., Heo J.Y., Lee M.E., Kim H.M., Paik S.G., Lee H., Lee J.O. (2007). Crystal structure of the TLR1-TLR2 heterodimer induced by binding of a tri-acylated lipopeptide. Cell.

[64] Park B.S., Song D.H., Kim H.M., Choi B.S., Lee H., Lee J.O. (2009). The structural basis of lipopolysaccharide recognition by the TLR4-MD-2 complex. Nature.

[65] Ohto U., Ishida H., Shibata T., Sato R., Miyake K., Shimizu T. (2018). Toll-like Receptor 9 Contains Two DNA Binding Sites that Function Cooperatively to Promote Receptor Dimerization and Activation. Immunity.

[66] Vasselon T., Detmers P.A., Charron D., Haziot A. (2004). TLR2 recognizes a bacterial lipopeptide through direct binding. J. Immunol..

[67] Raetz C.R., Reynolds C.M., Trent M.S., Bishop R.E. (2007). Lipid A modification systems in gram-negative bacteria. Annu. Rev. Biochem..

[68] Trent M.S., Stead C.M., Tran A.X., Hankins J.V. (2006). Diversity of endotoxin and its impact on pathogenesis. J. Endotoxin Res..

[69] Clementz T., Bednarski J.J., Raetz C.R. (1996). Function of the htrB high temperature requirement gene of *Escherichia coli* in the acylation of lipid A: HtrB catalyzed incorporation of laurate. J. Biol. Chem..

[70] Clementz T., Zhou Z., Raetz C.R. (1997). Function of the *Escherichia coli* msbB gene, a multicopy suppressor of htrB knockouts, in the acylation of lipid A. Acylation by MsbB follows laurate incorporation by HtrB. J. Biol. Chem..

[71] Bishop R.E., Kim S.H., El Zoeiby A. (2005). Role of lipid A palmitoylation in bacterial pathogenesis. J. Endotoxin Res..

[72] Reynolds C.M., Ribeiro A.A., McGrath S.C., Cotter R.J., Raetz C.R., Trent M.S. (2006). An outer membrane enzyme encoded by *Salmonella typhimurium* lpxR that removes the 3′-acyloxyacyl moiety of lipid A. J. Biol. Chem..

[73] Kawasaki K., Teramoto M., Tatsui R., Amamoto S. (2012). Lipid A 3′-O-deacylation by Salmonella outer membrane enzyme LpxR modulates the ability of lipid A to stimulate Toll-like receptor 4. Biochem. Biophys. Res. Commun..

[74] Lee H., Hsu F.F., Turk J., Groisman E.A. (2004). The PmrA-regulated pmrC gene mediates phosphoethanolamine modification of lipid A and polymyxin resistance in *Salmonella enterica*. J. Bacteriol..

[75] Kong Q., Six D.A., Roland K.L., Liu Q., Gu L., Reynolds C.M., Wang X., Raetz C.R., Curtiss R. (2011). Salmonella synthesizing 1-dephosphorylated [corrected] lipopolysaccharide exhibits low endotoxic activity while retaining its immunogenicity. J. Immunol..

[76] Anandan A., Vrielink A. (2020). Structure and function of lipid A–modifying enzymes. Ann. N. Y. Acad. Sci..

[77] Vorachek-Warren M.K., Carty S.M., Lin S., Cotter R.J., Raetz C.R. (2002). An *Escherichia coli* mutant lacking the cold shock-induced palmitoleoyltransferase of lipid A biosynthesis: Absence of unsaturated acyl chains and antibiotic hypersensitivity at 12 degrees C. J. Biol. Chem..

[78] Needham B.D., Carroll S.M., Giles D.K., Georgiou G., Whiteley M., Trent M.S. (2013). Modulating the innate immune response by combinatorial engineering of endotoxin. Proc. Natl. Acad. Sci. USA.

[79] Gioannini T.L., Teghanemt A., Zhang D., Coussens N.P., Dockstader W., Ramaswamy S., Weiss J.P. (2004). Isolation of an endotoxin-MD-2 complex that produces Toll-like receptor 4-dependent cell activation at picomolar concentrations. Proc. Natl. Acad. Sci. USA.

[80] Akashi S., Ogata H., Kirikae F., Kirikae T., Kawasaki K., Nishijima M., Shimazu R., Nagai Y., Fukudome K., Kimoto M. (2000). Regulatory roles for CD14 and phosphatidylinositol in the signaling via toll-like receptor 4-MD-2. Biochem. Biophys. Res. Commun..

[81] Rossi O., Pesce I., Giannelli C., Aprea S., Caboni M., Citiulo F., Valentini S., Ferlenghi I., MacLennan C.A., D’Oro U. (2014). Modulation of endotoxicity of *Shigella* generalized modules for membrane antigens (GMMA) by genetic lipid A modifications: Relative activation of TLR4 and TLR2 pathways in different mutants. J. Biol. Chem..

[82] Leitner D.R., Feichter S., Schild-Prüfert K., Rechberger G.N., Reidl J., Schild S. (2013). Lipopolysaccharide modifications of a cholera vaccine candidate based on outer membrane vesicles reduce endotoxicity and reveal the major protective antigen. Infect. Immun..

[83] Keiser P.B., Biggs-Cicatelli S., Moran E.E., Schmiel D.H., Pinto V.B., Burden R.E., Miller L.B., Moon J.E., Bowden R.A., Cummings J.F. (2011). A phase 1 study of a meningococcal native outer membrane vesicle vaccine made from a group B strain with deleted lpxL1 and synX, over-expressed factor H binding protein, two PorAs and stabilized OpcA expression. Vaccine.

[84] Keiser P.B., Gibbs B.T., Coster T.S., Moran E.E., Stoddard M.B., Labrie J.E., Schmiel D.H., Pinto V., Chen P., Zollinger W.D. (2010). A phase 1 study of a group B meningococcal native outer membrane vesicle vaccine made from a strain with deleted lpxL2 and synX and stable expression of opcA. Vaccine.

[85] Fransen F., Boog C.J., van Putten J.P., van der Ley P. (2007). Agonists of Toll-Like Receptors 3, 4, 7, and 9 Are Candidates for Use as Adjuvants in an Outer Membrane Vaccine against *Neisseria meningitidis* Serogroup B. Infect. Immun..

[86] Opal S.M., Palardy J.E., Chen W.H., Parejo N.A., Bhattacharjee A.K., Cross A.S. (2005). Active immunization with a detoxified endotoxin vaccine protects against lethal polymicrobial sepsis: Its use with CpG adjuvant and potential mechanisms. J. Infect. Dis..

[87] Bauman S.J., Kuehn M.J. (2006). Purification of outer membrane vesicles from *Pseudomonas aeruginosa* and their activation of an IL-8 response. Microbes Infect..

[88] Maggiore L., Yu L., Omasits U., Rossi O., Dougan G., Thomson N.R., Saul A., Choudhary J.S., Gerke C. (2016). Quantitative proteomic analysis of *Shigella flexneri* and *Shigella sonnei* Generalized Modules for Membrane Antigens (GMMA) reveals highly pure preparations. Int. J. Med. Microbiol..

[89] Nussenzweig M.C., Steinman R.M. (1980). Contribution of dendritic cells to stimulation of the murine syngeneic mixed leukocyte reaction. J. Exp. Med..

[90] Duthie M.S., Windish H.P., Fox C.B., Reed S.G. (2011). Use of defined TLR ligands as adjuvants within human vaccines. Immunol. Rev..

[91] Gnjatic S., Sawhney N.B., Bhardwaj N. (2010). Toll-like receptor agonists: Are they good adjuvants?. Cancer J..

[92] Turley S.J., Inaba K., Garrett W.S., Ebersold M., Unternaehrer J., Steinman R.M., Mellman I. (2000). Transport of peptide-MHC class II complexes in developing dendritic cells. Science.

[93] Blander J.M., Medzhitov R. (2006). Toll-dependent selection of microbial antigens for presentation by dendritic cells. Nature.

[94] Trombetta E.S., Ebersold M., Garrett W., Pypaert M., Mellman I. (2003). Activation of lysosomal function during dendritic cell maturation. Science.

[95] Nair-Gupta P., Baccarini A., Tung N., Seyffer F., Florey O., Huang Y., Banerjee M., Overholtzer M., Roche P.A., Tampé R. (2014). TLR signals induce phagosomal MHC-I delivery from the endosomal recycling compartment to allow cross-presentation. Cell.

[96] Kaisho T., Akira S. (2002). Toll-like receptors as adjuvant receptors. Biochim. Biophys. Acta.

[97] Ismail S., Hampton M.B., Keenan J.I. (2003). *Helicobacter pylori* Outer Membrane Vesicles Modulate Proliferation and Interleukin-8 Production by Gastric Epithelial Cells. Infect. Immun..

[98] Edwards A.D., Diebold S.S., Slack E.M.C., Tomizawa H., Hemmi H., Kaisho T., Akira S., Reis e Sousa C. (2003). Toll-like receptor expression in murine DC subsets: Lack of TLR7 expresion of CD8α+ DC correlates with unresponsiveness to imidazoquinolines. Eur. J. Immunol..

[99] Hornung V., Rothenfusser S., Britsch S., Krug A., Jahrsdörfer B., Giese T., Endres S., Hartmann G. (2002). Quantitative Expression of Toll-Like Receptor 1–10 mRNA in Cellular Subsets of Human Peripheral Blood Mononuclear Cells and Sensitivity to CpG Oligodeoxynucleotides. J. Immunol..

[100] Merad M., Sathe P., Helft J., Miller J., Mortha A. (2013). The Dendritic Cell Lineage: Ontogeny and Function of Dendritic Cells and Their Subsets in the Steady State and the Inflamed Setting. Annu. Rev. Immunol..

[101] Anderson D.A., Murphy K.M., Alt F. (2019). Chapter Four - Models of dendritic cell development correlate ontogeny with function. Advances in Immunology.

[102] Schulz O., Diebold S.S., Chen M., Näslund T.I., Nolte M.A., Alexopoulou L., Azuma Y.-T., Flavell R.A., Liljeström P., Reis e Sousa C. (2005). Toll-like receptor 3 promotes cross-priming to virus-infected cells. Nature.

[103] Browne E.P. (2012). Regulation of B-cell responses by Toll-like receptors. Immunology.

[104] Visintin A., Mazzoni A., Spitzer J.H., Wyllie D.H., Dower S.K., Segal D.M. (2001). Regulation of Toll-Like Receptors in Human Monocytes and Dendritic Cells. J. Immunol..

[105] Schreibelt G., Tel J., Sliepen K.H.E.W.J., Benitez-Ribas D., Figdor C.G., Adema G.J., de Vries I.J.M. (2010). Toll-like receptor expression and function in human dendritic cell subsets: Implications for dendritic cell-based anti-cancer immunotherapy. Cancer Immunol. Immunother..

[106] Komai-Koma M., Jones L., Ogg G.S., Xu D., Liew F.Y. (2004). TLR2 is expressed on activated T cells as a costimulatory receptor. Proc. Natl. Acad. Sci. USA.

[107] Kokkinopoulos I., Jordan W.J., Ritter M.A. (2005). Toll-like receptor mRNA expression patterns in human dendritic cells and monocytes. Mol. Immunol..

[108] Wesch D., Beetz S., Oberg H.H., Marget M., Krengel K., Kabelitz D. (2006). Direct costimulatory effect of TLR3 ligand poly(I:C) on human gamma delta T lymphocytes. J. Immunol..

[109] Reynolds J.M., Martinez G.J., Chung Y., Dong C. (2012). Toll-like receptor 4 signaling in T cells promotes autoimmune inflammation. Proc. Natl. Acad. Sci. USA.

[110] Bekeredjian-Ding I., Jego G. (2009). Toll-like receptors--sentries in the B-cell response. Immunology.

[111] Crellin N.K., Garcia R.V., Hadisfar O., Allan S.E., Steiner T.S., Levings M.K. (2005). Human CD4+ T cells express TLR5 and its ligand flagellin enhances the suppressive capacity and expression of FOXP3 in CD4+CD25+ T regulatory cells. J. Immunol..

[112] De Marcken M., Dhaliwal K., Danielsen A.C., Gautron A.S., Dominguez-Villar M. (2019). TLR7 and TLR8 activate distinct pathways in monocytes during RNA virus infection. Sci. Signal..

[113] Caron G., Duluc D., Frémaux I., Jeannin P., David C., Gascan H., Delneste Y. (2005). Direct Stimulation of Human T Cells via TLR5 and TLR7/8: Flagellin and R-848 Up-Regulate Proliferation and IFN-γ Production by Memory CD4^+^ T Cells. J. Immunol..

[114] Babu S., Blauvelt C.P., Kumaraswami V., Nutman T.B. (2006). Cutting Edge: Diminished T Cell TLR Expression and Function Modulates the Immune Response in Human Filarial Infection. J. Immunol..

[115] Jin B., Sun T., Yu X.-H., Yang Y.-X., Yeo A.E.T. (2012). The effects of TLR activation on T-cell development and differentiation. Clin. Dev. Immunol..

[116] Conroy H., Marshall N.A., Mills K.H. (2008). TLR ligand suppression or enhancement of Treg cells? A double-edged sword in immunity to tumours. Oncogene.

[117] Keijzer C., van der Zee R., van Eden W., Broere F. (2013). Treg inducing adjuvants for therapeutic vaccination against chronic inflammatory diseases. Front. Immunol..

[118] Toussi D.N., Massari P. (2014). Immune Adjuvant Effect of Molecularly-defined Toll-Like Receptor Ligands. Vaccines (Basel).

[119] Kreuk L.S., Koch M.A., Slayden L.C., Lind N.A., Chu S., Savage H.P., Kantor A.B., Baumgarth N., Barton G.M. (2019). B cell receptor and Toll-like receptor signaling coordinate to control distinct B-1 responses to both self and the microbiota. Elife.

[120] Pasare C., Medzhitov R. (2005). Control of B-cell responses by Toll-like receptors. Nature.

[121] Steinhagen F., Kinjo T., Bode C., Klinman D.M. (2011). TLR-based immune adjuvants. Vaccine.

[122] Boland G., Beran J., Lievens M., Sasadeusz J., Dentico P., Nothdurft H., Zuckerman J.N., Genton B., Steffen R., Loutan L. (2004). Safety and immunogenicity profile of an experimental hepatitis B vaccine adjuvanted with AS04. Vaccine.

[123] Wille-Reece U., Flynn B.J., Loré K., Koup R.A., Kedl R.M., Mattapallil J.J., Weiss W.R., Roederer M., Seder R.A. (2005). HIV Gag protein conjugated to a Toll-like receptor 7/8 agonist improves the magnitude and quality of Th1 and CD8+ T cell responses in nonhuman primates. Proc. Natl. Acad. Sci. USA.

[124] Nair S., McLaughlin C., Weizer A., Su Z., Boczkowski D., Dannull J., Vieweg J., Gilboa E. (2003). Injection of Immature Dendritic Cells into Adjuvant-Treated Skin Obviates the Need for Ex Vivo Maturation. J. Immunol..

[125] Mancini F., Monaci E., Lofano G., Torre A., Bacconi M., Tavarini S., Sammicheli C., Arcidiacono L., Galletti B., Laera D. (2016). One Dose of *Staphylococcus aureus* 4C-Staph Vaccine Formulated with a Novel TLR7-Dependent Adjuvant Rapidly Protects Mice through Antibodies, Effector CD4+ T Cells, and IL-17A. PLoS ONE.

[126] Schulze H.J., Cribier B., Requena L., Reifenberger J., Ferrándiz C., Garcia Diez A., Tebbs V., McRae S. (2005). Imiquimod 5% cream for the treatment of superficial basal cell carcinoma: Results from a randomized vehicle-controlled phase III study in Europe. Br. J. Dermatol..

[127] Lebwohl M., Dinehart S., Whiting D., Lee P.K., Tawfik N., Jorizzo J., Lee J.H., Fox T.L. (2004). Imiquimod 5% cream for the treatment of actinic keratosis: Results from two phase III, randomized, double-blind, parallel group, vehicle-controlled trials. J. Am. Acad. Dermatol..

[128] Feyerabend S., Stevanovic S., Gouttefangeas C., Wernet D., Hennenlotter J., Bedke J., Dietz K., Pascolo S., Kuczyk M., Rammensee H.G. (2009). Novel multi-peptide vaccination in Hla-A2+ hormone sensitive patients with biochemical relapse of prostate cancer. Prostate.

[129] Smorlesi A., Papalini F., Orlando F., Donnini A., Re F., Provinciali M. (2005). Imiquimod and S-27609 as adjuvants of DNA vaccination in a transgenic murine model of HER2/neu-positive mammary carcinoma. Gene Ther..

[130] Adams S., O’Neill D.W., Nonaka D., Hardin E., Chiriboga L., Siu K., Cruz C.M., Angiulli A., Angiulli F., Ritter E. (2008). Immunization of malignant melanoma patients with full-length NY-ESO-1 protein using TLR7 agonist imiquimod as vaccine adjuvant. J. Immunol..

[131] Kim O.Y., Hong B.S., Park K.S., Yoon Y.J., Choi S.J., Lee W.H., Roh T.Y., Lötvall J., Kim Y.K., Gho Y.S. (2013). Immunization with *Escherichia coli* outer membrane vesicles protects bacteria-induced lethality via Th1 and Th17 cell responses. J. Immunol..

[132] Lee B.-J., Kwon H.-I., Kim E.-H., Park S.-J., Lee S.-H., Choi Y.K., Kim S.-H. (2014). Assessment of mOMV adjuvant efficacy in the pathogenic H1N1 influenza virus vaccine. Clin. Exp. Vaccine Res..

[133] Lee T.Y., Kim C.U., Bae E.H., Seo S.H., Jeong D.G., Yoon S.W., Chang K.T., Kim Y.S., Kim S.H., Kim D.J. (2017). Outer membrane vesicles harboring modified lipid A moiety augment the efficacy of an influenza vaccine exhibiting reduced endotoxicity in a mouse model. Vaccine.

[134] Holst J., Martin D., Arnold R., Huergo C.C., Oster P., O’Hallahan J., Rosenqvist E. (2009). Properties and clinical performance of vaccines containing outer membrane vesicles from *Neisseria meningitidis*. Vaccine.

[135] Frasch C.E. (1990). Production and control of *Neisseria meningitidis* vaccines. Adv. Biotechnol. Process..

[136] Durand V., MacKenzie J., de Leon J., Mesa C., Quesniaux V., Montoya M., Le Bon A., Wong S.Y.C. (2009). Role of lipopolysaccharide in the induction of type I interferon-dependent cross-priming and IL-10 production in mice by meningococcal outer membrane vesicles. Vaccine.

[137] Schild S., Nelson E.J., Camilli A. (2008). Immunization with *Vibrio cholerae* Outer Membrane Vesicles Induces Protective Immunity in Mice. Infect. Immun..

[138] Alaniz R.C., Deatherage B.L., Lara J.C., Cookson B.T. (2007). Membrane Vesicles Are Immunogenic Facsimiles of *Salmonella typhimurium* That Potently Activate Dendritic Cells, Prime B and T Cell Responses, and Stimulate Protective Immunity In Vivo. J. Immunol..

[139] Whitmire W.M., Garon C.F. (1993). Specific and nonspecific responses of murine B cells to membrane blebs of *Borrelia burgdorferi*. Infect. Immun..

[140] Aoki M., Kondo M., Nakatsuka Y., Kawai K., Oshima S. (2007). Stationary phase culture supernatant containing membrane vesicles induced immunity to rainbow trout *Oncorhynchus mykiss* fry syndrome. Vaccine.

[141] Arigita C., Kersten G.F., Hazendonk T., Hennink W.E., Crommelin D.J., Jiskoot W. (2003). Restored functional immunogenicity of purified meningococcal PorA by incorporation into liposomes. Vaccine.

[142] Marini A., Rossi O., Aruta M.G., Micoli F., Rondini S., Guadagnuolo S., Delany I., Henderson I.R., Cunningham A.F., Saul A. (2017). Contribution of factor H-Binding protein sequence to the cross-reactivity of meningococcal native outer membrane vesicle vaccines with over-expressed fHbp variant group 1. PLoS ONE.

[143] Raeven R.H., van der Maas L., Tilstra W., Uittenbogaard J.P., Bindels T.H., Kuipers B., van der Ark A., Pennings J.L., van Riet E., Jiskoot W. (2015). Immunoproteomic Profiling of Bordetella pertussis Outer Membrane Vesicle Vaccine Reveals Broad and Balanced Humoral Immunogenicity. J. Proteome Res..

[144] Gaillard M.E., Bottero D., Errea A., Ormazábal M., Zurita M.E., Moreno G., Rumbo M., Castuma C., Bartel E., Flores D. (2014). Acellular pertussis vaccine based on outer membrane vesicles capable of conferring both long-lasting immunity and protection against different strain genotypes. Vaccine.

[145] Muralinath M., Kuehn M.J., Roland K.L., Curtiss R. (2011). Immunization with *Salmonella enterica* Serovar Typhimurium-Derived Outer Membrane Vesicles Delivering the Pneumococcal Protein PspA Confers Protection against Challenge with *Streptococcus pneumoniae*. Infect. Immun..

[146] Huang W., Wang S., Yao Y., Xia Y., Yang X., Li K., Sun P., Liu C., Sun W., Bai H. (2016). Employing *Escherichia coli*-derived outer membrane vesicles as an antigen delivery platform elicits protective immunity against *Acinetobacter baumannii* infection. Sci. Rep..

[147] Chen L., Valentine J.L., Huang C.-J., Endicott C.E., Moeller T.D., Rasmussen J.A., Fletcher J.R., Boll J.M., Rosenthal J.A., Dobruchowska J. (2016). Outer membrane vesicles displaying engineered glycotopes elicit protective antibodies. Proc. Natl. Acad. Sci. USA.

[148] Bartolini E., Ianni E., Frigimelica E., Petracca R., Galli G., Berlanda Scorza F., Norais N., Laera D., Giusti F., Pierleoni A. (2013). Recombinant outer membrane vesicles carrying *Chlamydia muridarum* HtrA induce antibodies that neutralize chlamydial infection in vitro. J. Extracell. Vesicles.

[149] Fantappiè L., de Santis M., Chiarot E., Carboni F., Bensi G., Jousson O., Margarit I., Grandi G. (2014). Antibody-mediated immunity induced by engineered *Escherichia coli* OMVs carrying heterologous antigens in their lumen. J. Extracell. Vesicles.

[150] Chen D.J., Osterrieder N., Metzger S.M., Buckles E., Doody A.M., DeLisa M.P., Putnam D. (2010). Delivery of foreign antigens by engineered outer membrane vesicle vaccines. Proc. Natl. Acad. Sci. USA.

[151] Valguarnera E., Feldman M.F. (2017). Glycoengineered Outer Membrane Vesicles as a Platform for Vaccine Development. Methods Enzymol..

[152] Price N.L., Goyette-Desjardins G., Nothaft H., Valguarnera E., Szymanski C.M., Segura M., Feldman M.F. (2016). Glycoengineered Outer Membrane Vesicles: A Novel Platform for Bacterial Vaccines. Sci. Rep..

[153] Fransen F., Stenger R.M., Poelen M.C.M., van Dijken H.H., Kuipers B., Boog C.J.P., van Putten J.P.M., van Els C.A.C.M., van der Ley P. (2010). Differential effect of TLR2 and TLR4 on the immune response after immunization with a vaccine against *Neisseria meningitidis* or *Bordetella pertussis*. PLoS ONE.

[154] Wu Y., Przysiecki C., Flanagan E., Bello-Irizarry S.N., Ionescu R., Muratova O., Dobrescu G., Lambert L., Keister D., Rippeon Y. (2006). Sustained high-titer antibody responses induced by conjugating a malarial vaccine candidate to outer-membrane protein complex. Proc. Natl. Acad. Sci. USA.

[155] Latz E., Franko J., Golenbock D.T., Schreiber J.R. (2004). *Haemophilus influenzae* Type b-Outer Membrane Protein Complex Glycoconjugate Vaccine Induces Cytokine Production by Engaging Human Toll-Like Receptor 2 (TLR2) and Requires the Presence of TLR2 for Optimal Immunogenicity. J. Immunol..

[156] Bishop R.E. (2016). Polymorphic Regulation of Outer Membrane Lipid A Composition. mBio.

[157] Rivero-Calle I., Raguindin P.F., Gómez-Rial J., Rodriguez-Tenreiro C., Martinón-Torres F. (2019). Meningococcal Group B Vaccine For The Prevention Of Invasive Meningococcal Disease Caused By *Neisseria meningitidis* Serogroup B. Infect. Drug Resist..

[158] Obiero C.W., Ndiaye A.G.W., Sciré A.S., Kaunyangi B.M., Marchetti E., Gone A.M., Schütte L.D., Riccucci D., Auerbach J., Saul A. (2017). A Phase 2a Randomized Study to Evaluate the Safety and Immunogenicity of the 1790GAHB Generalized Modules for Membrane Antigen Vaccine against *Shigella sonnei* Administered Intramuscularly to Adults from a Shigellosis-Endemic Country. Front. Immunol..

[159] Li Y.P., Yu S.L., Huang Z.J., Huang J., Pan J., Feng X., Zhang X.G., Wang J.H., Wang J. (2015). An impaired inflammatory cytokine response to gram-negative LPS in human neonates is associated with the defective TLR-mediated signaling pathway. J. Clin. Immunol..

[160] Grabiec A., Meng G., Fichte S., Bessler W., Wagner H., Kirschning C.J. (2004). Human but not murine toll-like receptor 2 discriminates between tri-palmitoylated and tri-lauroylated peptides. J. Biol. Chem..

[161] Jurk M., Heil F., Vollmer J., Schetter C., Krieg A.M., Wagner H., Lipford G., Bauer S. (2002). Human TLR7 or TLR8 independently confer responsiveness to the antiviral compound R-848. Nat. Immunol..

[162] Bauer S., Kirschning C.J., Häcker H., Redecke V., Hausmann S., Akira S., Wagner H., Lipford G.B. (2001). Human TLR9 confers responsiveness to bacterial DNA via species-specific CpG motif recognition. Proc. Natl. Acad. Sci. USA.

[163] Chuang T.H., Lee J., Kline L., Mathison J.C., Ulevitch R.J. (2002). Toll-like receptor 9 mediates CpG-DNA signaling. J. Leukoc. Biol..

